# Fracture of Two Moderately Cross-Linked Polyethylene Tibial Inserts in a TKR Patient

**DOI:** 10.1155/2014/491384

**Published:** 2014-01-08

**Authors:** Matthew G. Teeter, James P. McAuley, Douglas D. Naudie

**Affiliations:** Division of Orthopaedic Surgery, London Health Sciences Centre-University Hospital, 339 Windermere Road London, ON, Canada N6A 5A5

## Abstract

Highly cross-linked polyethylene has become the gold standard in total hip replacement for its wear resistance. Moderately crosslinked polyethylene is now available for total knee replacement (TKR), although concerns about reduced mechanical strength have prevented widespread adoption. The purpose of this report is to describe an unusual case where a patient underwent cruciate retaining TKR using a moderately crosslinked polyethylene tibial insert that went on to fracture twice in the same location across the primary and first revision surgery. The first tibial insert was 10 mm thick and was implanted for 16 months. The second tibial insert was 15 mm thick and was implanted for 11 months. Both fractured along the posterior aspect of the medial articular surface. The lack of a specific event leading to these fractures and the fact that they occurred twice in the same location in the same patient suggest that caution is still necessary regarding the introduction of crosslinked polyethylene for TKR surgery.

## 1. Introduction

Highly cross-linked polyethylene was introduced for THR in the last decade and has since demonstrated excellent long-term wear resistance, leading to its acceptance as the new clinical standard for THR implants [1]. Adoption of cross linking for TKR has been more controversial, as the cross linking process can decrease the mechanical properties of the polyethylene in addition to increasing its wear resistance [2]. In *in vitro* testing, TKR implants made with moderate cross linking of the polyethylene have shown decreased wear and similar mechanical strength compared to implants using conventional polyethylene, and on this basis moderately cross-linked polyethylene inserts have been introduced for clinical use [3–5]. Most early clinical reports of cross-linked polyethylene in TKR report favorable results, with good wear resistance [6]. However, there have been reports of fractures of highly cross-linked polyethylene in TKR, with all-polyethylene patellar components, at the tibial post of posterior stabilized tibial inserts, and across the condylar surface of CR inserts [7–11].

The purpose of this report is to describe an unusual case in which a patient had a fracture of the moderately cross-linked polyethylene tibial insert of their total knee replacement, at the same posteromedial articular surface location, across two different surgeries in the same joint, within a short period of implantation.

## 2. Case Presentation

The patient was a woman who underwent primary TKA surgery in October 2010 on her left knee, for osteoarthritis involving the medial tibiofemoral and patellofemoral compartments. At that time she was 61 years of age, approximately 156 cm tall and weighed 90.5 kg, with a body mass index (BMI) of 36.7 kg/m^2^. The treating surgeon implanted a Sigma fixed bearing, cruciate retaining (CR) knee system (Depuy Synthes, Warsaw, IN, USA), consisting of a size 3 CR left femoral component, size 2 tibial tray, and a 10 mm thick, size 2 moderately cross-linked XLK polyethylene posteriorly lipped CR tibial insert. A posteromedial release of the soft tissues was performed, which with these components resulted in excellent symmetrical opening of 1 to 2 mm medially and laterally at 90 degrees flexion and at full extension, tibiofemoral contact at the midpoint of the tibia at 90 degrees, and excellent posterior cruciate ligament (PCL) function with no signs of insufficiency or tightness. The patient had an approximate flexion range of 0 to 115 degrees following the operation.

**Figure 1:**
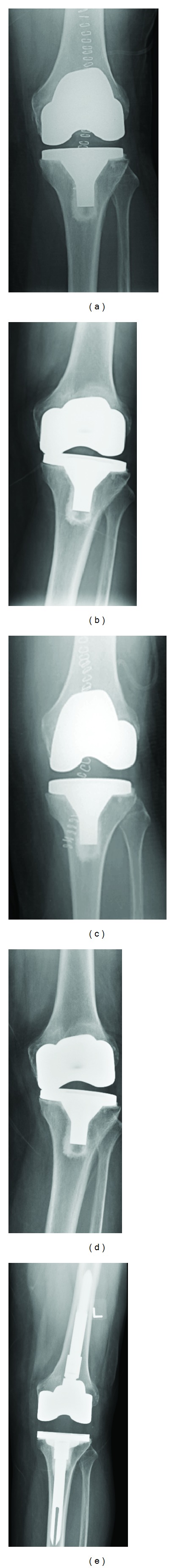
Anterior-posterior radiographs of the left knee. (a) After the primary surgery. (b) The failed primary tibial insert, before the first revision surgery. (c) After the first revision surgery. (d) The failed revision tibial insert before the second revision surgery. (e) After the second revision surgery, in which all of the components were removed and a new posterior-stabilized implant system was used.

The patient was satisfied with the surgery until December 2011, at which time she developed pain and a clunking sensation of instability in her surgical knee that came on quite suddenly. No incident preceded this sensation. She also complained of activity related swelling and had a varus thrust while walking. Failure of the polyethylene tibial insert was suspected (Figure 1(a)), and the patient underwent revision surgery in early February 2012, approximately 1 year and 4 months after her primary surgery. Under general anesthesia her knee appeared to demonstrate good stability, but there was a palpable grating sensation throughout passive motion from 0 to 110 degrees. Upon opening the joint, a marked mechanical failure of the posteromedial aspect of the polyethylene was evident. The locking mechanism was unaffected. Some scratching on the medial aspect of the femoral component was present due to wear-through of the polyethylene; however, both the femoral and tibial components were well fixed and were left in place. A new 10 mm thick tibial insert was trialed; however, this was found to be too loose in extension. A thicker tibial insert was then trialed but was too tight in flexion, secondary to a tight PCL. A PCL release was performed and a 15 mm thick tibial insert was again trialed. The flexion space was felt to be 1 to 2 mm symmetrically at  90 degrees, with appropriate midpoint contact, and a drop flexion of 115 to 120 degrees from full extension. A moderately cross-linked CR tibial insert was once again used.

The patient was once again satisfied with the revision operation to her left knee, and underwent primary TKA surgery to her right knee in June 2012. In light of her issues on the left side, a posterior stabilized, conventional polyethylene version of the Sigma knee system was used and has performed well since that time. In September 2012, the patient again became bothered with her left knee, with the feeling of giving way, especially on stairs. She demonstrated significant anterior-posterior and medial-lateral instability, and a second fracture of the tibial polyethylene insert was suspected on radiographs (Figure 1(b)). The patient underwent a second revision surgery in January 2013 under a second surgeon, approximately 11 months following her last revision surgery (Figure 1(c)). Fracture of the posteromedial aspect of the polyethylene tibial insert was again seen upon opening the joint capsule. There was no obvious ligament imbalance of the knee and no malposition of the femoral or tibial components. The knee components were well fixed, but it was decided that a complete revision of her implants was required, and a Legion revision knee system was employed (Smith  &  Nephew, Memphis, TN). On the femoral side, this consisted of a left size 4 constrained femoral component, a 14 mm × 160 mm straight press fit stem, a 4 mm posterior couple, a 10 mm distal lateral augment, and a 5 mm distal medial augment. On the tibial side, this consisted of a left size 2 tibial component, 13 mm × 160 mm straight stem, and a size 1-2 18 mm thick high flexion posterior stabilized Genesis II tibial insert made from conventional, non-cross-linked polyethylene. The patella was resurfaced at the time of this revision arthroplasty. At a 6-week followup she reports no pain, has a good range of motion from 0 to 110 degrees, and reports being happier with her twice-revised left knee than with the right knee.

**Figure 2:**
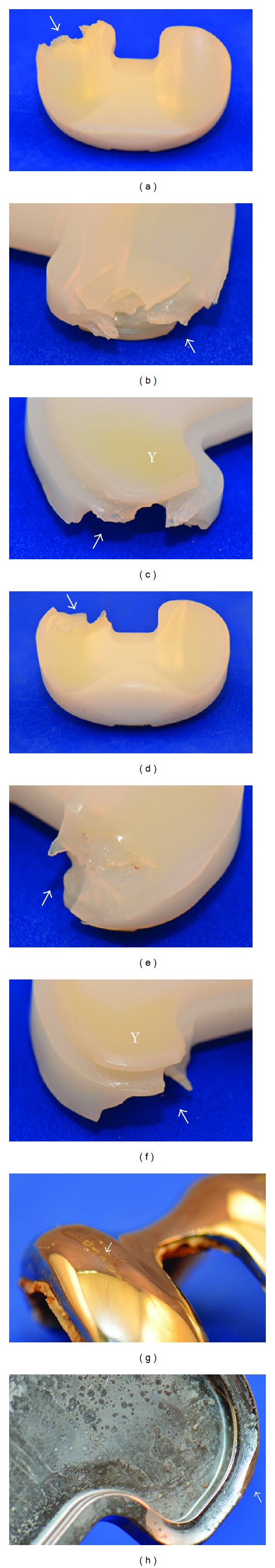
Photographs of damage to the implanted components. (a) Tibial insert from the primary surgery, with the fracture on the medial side (arrow). (b) Close-up view of the fracture (arrow) on the articular surface. (c) Close-up view of the backside opposite the fracture (arrow), with yellow discoloration (Y). (d) Tibial insert from the first revision surgery, with the fracture in the same spot on the medial side (arrow). (e) Close-up view of the fracture (arrow) on the articular surface. (f) Close-up view of the backside opposite the fracture (arrow), with yellow discoloration (Y). (g) Damage (scratches and pits, arrow) to the medial side of the femoral component. (h) Damage (arrow) along the edge of the medial side of the tibial baseplate.

Each retrieved component was examined and photographed (Figure 2). The tibial insert from the primary surgery demonstrated an extensive fracture at the posterior aspect of the medial side (Figures 2(a) and 2(b)). Embedded metal debris was also seen on the medial side, along with scratching, pitting, and burnishing. The lateral side demonstrated even greater scratching and pitting, along with burnishing, but no embedded debris. The backside was generally pristine with the exception of a few scratches at the posterior aspect of the medial side. A slight yellow hue was noted on the medial backside surface, below where the fracture had occurred (Figure 2(c)). The tibial insert from the revision surgery also demonstrated an extensive fracture at the same location as the insert from the primary surgery (Figures 2(d) and 2(e)). Much less scratching and pitting was seen on both the medial and lateral side of the articular surface in comparison to the primary tibial insert, with little burnishing and no embedded debris. The backside was again in good shape, with some scratching and pitting on the posterior aspect of the medial side. A yellow hue was again seen on the backside, along the posterolateral locking mechanism and especially on the medial side beneath the fracture (Figure 2(f)). On the femoral component, extensive scratches running the length of the medial side were observed (Figure 2(g)), with some minor scratches also on the lateral side. The tibial tray was in good shape overall, with some wear seen at the posterior edge of the locking mechanism on the medial side, where the femoral component likely articulated after the fracture of the polyethylene (Figure 2(h)).

**Figure 3:**
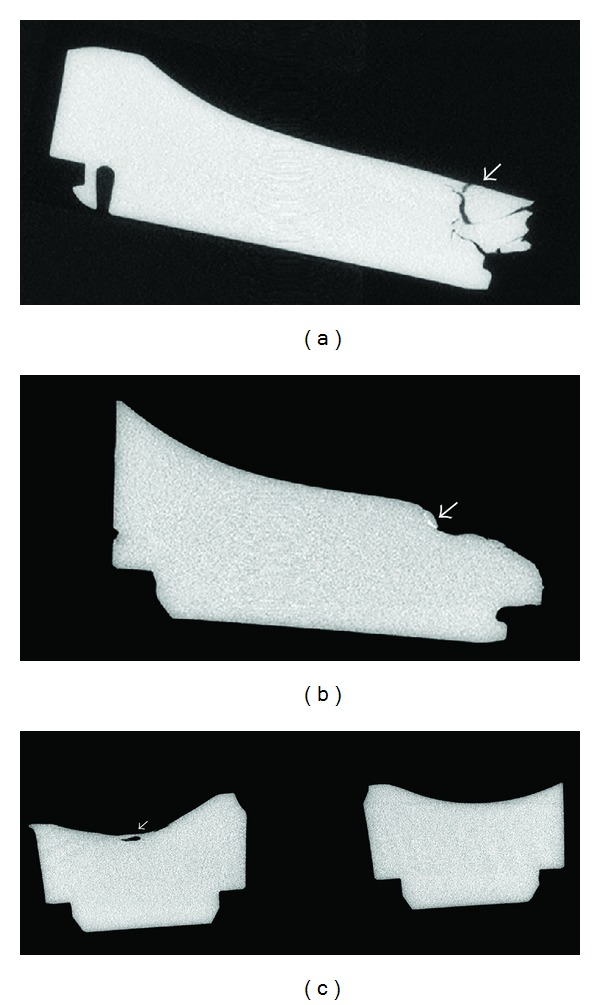
Micro-CT examination of the retrieved tibial inserts subsurface. (a) Medial-lateral view of the retrieved tibial insert from the primary surgery, demonstrating extensive cracks (arrow). (b) Medial-lateral view of the retrieved tibial insert from the first revision surgery, with a region of enhanced intensity near the fracture site (arrow). (c) Anterior-posterior view of the retrieved tibial insert from the first revision surgery, with a contained subsurface void (arrow).

To determine whether any further fatigue cracking had taken place, the tibial inserts from the primary and first revision surgery were scanned with a laboratory microcomputed tomography (micro-CT) scanner, in a manner described previously [10]. The reconstructed image volumes were examined for evidence of subsurface cracks. Cracks did extend a few millimeters further from the location of the fracture in the subsurface of both the primary and first revision tibial insert, beyond what could be appreciated from a standard visual inspection (Figure 3). A region of increased density was also seen within one of the subsurface cracks for the first revision tibial insert (Figure 3(b)). This has been thought to be associated with the early stages crack initiation from fatigue loading [10]. No further cracks were seen for the remainder of the tibial insert subsurface, outside of the fractured region.

## 3. Discussion

We have presented an unusual case, where a TKR implanted by an experienced surgeon fractured twice across two short implantation times at the same location within a moderately cross-linked polyethylene tibial insert. In both cases, the implants appeared well positioned, with good soft tissue balance at the time of the operation. However, at the time of the first revision surgery, there was evident soft tissue changes that required a further soft tissue release and the use of a thicker tibial insert, suggesting that the soft tissue may have become unbalanced over time. Some of these changes could also have occurred after the polyethylene fractured. In both cases, the relatively short implantation times (16 months and 11 months) suggest an unusual loading pattern may have occurred, rather than fatigue over a great number of loading cycles. No specific event (e.g. trauma or a fall) was reported by the patient to have preceded either polyethylene fracture. The patient did not take part in any unusual high demand activities outside of daily living. The patient would be classified as severely to very severely obese based on their BMI, which increased from 36.7 kg/m^2^ at the primary surgery to 40.3 kg/m^2^ at the time of the second revision; as such, this load could also be a factor.

Another area of suspicion is of course the polyethylene itself. XLK polyethylene was introduced by Depuy in 2005, and is fabricated from machined GUR 1020 ram extruded bar stock. Cross-linking is accomplished through 5 MRad of gamma radiation, followed by a melt-annealing process, with the polyethylene packed in a vacuum-sealed bag and sterilized using gas plasma. The melt annealing process has been demonstrated to impart greater wear and fatigue resistance with moderately to highly cross-linked polyethylene in comparison to conventional polyethylene [5]. The Sigma implant has been specifically studied under wear simulator testing, and the moderately cross-linked version demonstrated significantly less wear than the conventional polyethylene version [3]. The locking mechanism held up well, even with both fractures, in contrast to other cross-linked models that have had issues with locking mechanism dissociation [12]. The yellow hues seen in the retrieved tibial inserts might be associated with oxidation; however, we lacked the necessary equipment to measure oxidation within the polyethylene. Oxidation can severely limit the longevity of polyethylene, which has resulted in manufacturers developing methods to introduce antioxidants such as vitamin E into their polyethylene components, to stabilize their properties [4].

As with most cases of implant failure, a combination of patient, surgeon, and implant factors likely contributed to the fracture of the polyethylene tibial inserts seen with this case. The results demonstrate that even with apparently good initial surgical outcomes, negative results can occur. The lack of a specific event leading to these fractures and the fact that they occurred twice in the same location in the same patient suggest that caution is still necessary regarding the introduction of cross-linked polyethylene for TKR surgery. This particular implant has performed well in preclinical testing, providing further evidence that *in vitro* tests cannot always reproduce *in vivo* conditions.

## References

[1] Kurtz SM, Gawel HA, Patel JD (2011). History and systematic review of wear and osteolysis outcomes for first-generation highly crosslinked polyethylene. *Clinical Orthopaedics and Related Research*.

[2] Lachiewicz PF, Geyer MR (2011). The use of highly cross-linked polyethylene in total knee arthroplasty. *Journal of the American Academy of Orthopaedic Surgeons*.

[3] Fisher J, McEwen HMJ, Tipper JL (2004). Wear, debris, and biologic activity of cross-linked polyethylene in the knee: benefits and potential concerns. *Clinical Orthopaedics and Related Research*.

[4] Haider H, Weisenburger JN, Kurtz SM (2012). Does vitamin E-stabilized ultrahigh-molecular-weight polyethylene address concerns of cross-linked polyethylene in total knee arthroplasty?. *Journal of Arthroplasty*.

[5] Popoola OO, Yao JQ, Johnson TS, Blanchard CR (2010). Wear, delamination, and fatigue resistance of melt-annealed highly crosslinked UHMWPE cruciate-retaining knee inserts under activities of daily living. *Journal of Orthopaedic Research*.

[6] Teeter MG, Yuan X, Naudie DDR, Holdsworth DW (2010). Technique to quantify subsurface cracks in retrieved polyethylene components using micro-CT. *Journal of Long-Term Effects of Medical Implants*.

[7] Barrack RL (2013). Retrieval analysis of an early fracture of a vitamin E-stabilized tibial liner in total knee arthroplasty. *Case Connector*.

[8] Goldstein MJ, Ast MP, Dimaio FR (2012). Acute posttraumatic catastrophic failure of a second-generation, highly cross-linked ultra-high-molecular-weight polyethylene patellar component. *Orthopedics*.

[9] Hambright DS, Watters TS, Kaufman AM, Lachiewicz PF, Bolognesi MP (2010). Fracture of highly cross-linked all-polyethylene patella after total knee arthroplasty. *The journal of knee surgery*.

[10] Teeter MG, Yuan X, Naudie DDR, Holdsworth DW (2010). Technique to quantify subsurface cracks in retrieved polyethylene components using micro-CT. *Journal of Long-Term Effects of Medical Implants*.

[11] Yoon JR, Jeong HI, Oh KJ, Yang JH (2013). Bilateral condyle fracture of tibial insert in mobile bearing total knee arthroplasty. *Knee*.

[12] Willie BM, Foot LJ, Prall MW, Bloebaum RD (2008). Surface damage analysis of retrieved highly crosslinked polyethylene tibial components after short-term implantation. *Journal of Biomedical Materials Research B*.

